# Metabolic fingerprinting of systemic sclerosis: a systematic review

**DOI:** 10.3389/fmolb.2023.1215039

**Published:** 2023-08-08

**Authors:** Victoria Morales-González, Daniel Galeano-Sánchez, Jaime Enrique Covaleda-Vargas, Yhojan Rodriguez, Diana M. Monsalve, Daniel Pardo-Rodriguez, Mónica P. Cala, Yeny Acosta-Ampudia, Carolina Ramírez-Santana

**Affiliations:** ^1^ Center for Autoimmune Diseases Research (CREA), School of Medicine and Health Sciences, Universidad Del Rosario, Bogotá, Colombia; ^2^ Metabolomics Core Facility—MetCore, Vicepresidency for Research, Universidad de Los Andes, Bogotá, Colombia

**Keywords:** systemic sclerosis, metabolomics, metabolic pathways, biomarkers, amino acids

## Abstract

**Introduction:** Systemic sclerosis (SSc) is a chronic autoimmune disease, marked by an unpredictable course, high morbidity, and increased mortality risk that occurs especially in the diffuse and rapidly progressive forms of the disease, characterized by fibrosis of the skin and internal organs and endothelial dysfunction. Recent studies suggest that the identification of altered metabolic pathways may play a key role in understanding the pathophysiology of the disease. Therefore, metabolomics might be pivotal in a better understanding of these pathogenic mechanisms.

**Methods:** Through a systematic review of the literature following the Preferred Reporting Items for Systematic Reviews and Meta-Analyses Guidelines (PRISMA), searches were done in the PubMed, EMBASE, Web of Science, and Scopus databases from 2000 to September 2022. Three researchers independently reviewed the literature and extracted the data based on predefined inclusion and exclusion criteria.

**Results:** Of the screened studies, 26 fulfilled the inclusion criteria. A total of 151 metabolites were differentially distributed between SSc patients and healthy controls (HC). The main deregulated metabolites were those derived from amino acids, specifically homocysteine (Hcy), proline, alpha-N-phenylacetyl-L-glutamine, glutamine, asymmetric dimethylarginine (ADMA), citrulline and ornithine, kynurenine (Kyn), and tryptophan (Trp), as well as acylcarnitines associated with long-chain fatty acids and tricarboxylic acids such as citrate and succinate. Additionally, differences in metabolic profiling between SSc subtypes were identified. The diffuse cutaneous systemic sclerosis (dcSSc) subtype showed upregulated amino acid-related pathways involved in fibrosis, endothelial dysfunction, and gut dysbiosis. Lastly, potential biomarkers were evaluated for the diagnosis of SSc, the identification of the dcSSc subtype, pulmonary arterial hypertension, and interstitial lung disease. These potential biomarkers are within amino acids, nucleotides, carboxylic acids, and carbohydrate metabolism.

**Discussion:** The altered metabolite mechanisms identified in this study mostly point to perturbations in amino acid-related pathways, fatty acid beta-oxidation, and in the tricarboxylic acid cycle, possibly associated with inflammation, vascular damage, fibrosis, and gut dysbiosis. Further studies in targeted metabolomics are required to evaluate potential biomarkers for diagnosis, prognosis, and treatment response.

## 1 Introduction

Systemic sclerosis is a chronic and rare autoimmune disease of the connective tissue, whose etiology remains unknown, and the pathogenesis is still partially understood, thus representing a clinical challenge and an unmet medical need (Denton and Khanna, 2017; Tsou et al., 2021). SSc is characterized by a pathogenic triad consisting of microvascular damage, innate and adaptive immune system abnormalities with autoantibody production and cell-mediated autoimmunity, and fibroblast dysfunction with excessive collagen deposition, which leads to progressive fibrosis of the skin and internal organs (Allanore et al., 2015; Pattanaik et al., 2015).

A striking characteristic of the disease is the variability within patients, with great heterogeneity in the clinical manifestations, the serological profiles, and the disease progression rate. SSc is characterized by high morbidity and mortality, higher than any other rheumatic disease (Elhai et al., 2017). Worldwide, the prevalence of SSc is approximately 17.6 cases per 100,000 population, and the incidence rate is 1.4 per 100,000 person-years; however, there is great variability among geographic populations (Bairkdar et al., 2021). Recent studies have reported a mortality rate of 1.39–5.1 times higher than the general population (Elhai et al., 2012; Rubio-Rivas et al., 2014; Hao et al., 2017; Kang et al., 2018). SSc has a disease-related mortality rate of approximately 55%, with the leading causes of death being pulmonary complications such as interstitial lung disease (ILD), followed by pulmonary arterial hypertension (PAH) (Tyndall et al., 2010). ILD has a 19%–52% prevalence in SSc patients (Perelas et al., 2020; Kuwana et al., 2022), and approximately 40% of these patients die within 10 years of diagnosis (Akter et al., 2014). The pathogenesis of SSc-ILD begins with a permanent injury to the alveolar epithelium, secondary to an activation of the immune system promoting profibrotic stimuli that induce fibroblast recruitment and differentiation to a myofibroblast phenotype and extracellular matrix (ECM) overproduction (Nihtyanova and Denton, 2020). Moreover, in SSc patients, PAH occurs with a prevalence of 5%–15% (Morrisroe et al., 2017; Naranjo and Hassoun, 2021). PAH is characterized by arterial remodeling, and an increased pulmonary vascular resistance secondary to abnormal vascular proliferation, disequilibrium in vasodilators, proliferative mediators, and thrombosis of the pulmonary vasculature resulting in right heart failure, which can eventually lead to death (Launay et al., 2017).

In relation to skin involvement, SSc can be subclassified into diffuse cutaneous systemic sclerosis (dcSSc) and limited cutaneous systemic sclerosis (lcSSc) (Young and Khanna, 2015). LcSSc is the most frequent subtype presentation (Coral-Alvarado et al., 2009), characterized by a gradual and early onset of Raynaud’s phenomenon and skin fibrosis restricted to certain areas, such as the face and distal extremities, with minor systemic involvement (Herrick, 2018). In contrast, in the dcSSc subtype, Raynaud’s phenomenon coexists with skin fibrosis extended proximally to knees, elbows, and the trunk with a more aggressive progression characterized by severe internal organ manifestations, mainly in the gastrointestinal tract, lungs, heart, and kidneys (Kowalska-Kępczyńska, 2022).

A better understanding of the pathophysiology of SSc is crucial to tackle the processes leading to disease progression and to discover effective therapies to improve the long-term survival of SSc patients. Recent studies suggest that metabolic perturbations may play an important role in SSc pathogenesis and are exhibited in different patients as a result of the disease heterogeneity and erratic course (Wishart, 2016; Cambiaghi et al., 2017; O’Reilly, 2022). Therefore, metabolomics may play an important role in understanding the pathophysiology of the disease.

Metabolic characterization represents a promising approach that can be applied for diagnosis, disease typing, and individual treatment of SSc, as well as biomarker discovery (Zhang et al., 2015a). Thus, this review aimed to identify altered metabolic pathways possibly responsible for the mechanisms associated with the appearance of SSc, in order to improve diagnosis, prognosis, and treatment. Additionally, it identifies metabolites that allow the segregation of patients with dcSSc and lcSSc.

## 2 Methods

### 2.1 Information sources and search strategy

A systematic review of the literature was conducted following the recommendations of the PRISMA guidelines (Page et al., 2021). Published studies related to the topic were retrieved after a literature search in four databases: PubMed, EMBASE, Web of Science, and Scopus, from January 2000 to September 2022. The references listed in the articles were manually searched. Only English and Spanish language articles were included. A search strategy combining MESH terms and free words were developed: (“Metabolomics,” “Untargeted Metabolomics,” “Metabolomic Fingerprinting,” “Metabonomic,” “Targeted Metabolomics,” “Metabolic footprinting,” “Metabolic profiling,” “Metabolome,” “Metabolic profile,” “Lipidomics,” “Lipidome” OR “Lipidomes”) AND (“Scleroderma, Systemic,” “Scleroderma, Diffuse,” “Scleroderma, Limited,” “CREST Syndrome,” “Systemic Sclerosis,” “Systemic Scleroderma” OR “Sclerosis, Systemic”). Supplementary Table S1 depicts the search strategy 1. This systematic review was not registered. Protocol was not written prior to the elaboration of the systematic review.

### 2.2 Eligibility criteria

Studies meeting the following criteria were included: 1) analytical observational studies (i.e., cross-sectional, case-control, and cohort studies) that evaluated altered metabolites in biological samples such as serum, plasma, urine, and exhaled breath by high-throughput techniques in patients diagnosed with SSc in comparison to HC; 2) studies published in English and/or Spanish and 3) studies implemented in adults. The exclusion criteria were as follows: 1) animal or *in vitro* cell studies; 2) non-original articles; 3) conference abstracts, guidelines, or editorials; 4) studies using irrelevant metabolomics techniques and 5) article data incomplete or missing.

### 2.3 Study selection

Three reviewers (VM, JC, DG), after removing duplicate articles, independently reviewed all the selected studies in the initial research in a two-step procedure assessing their eligibility. In the first phase, all identified titles and abstracts were evaluated to determine which records were possibly eligible for inclusion. Subsequently, the potentially relevant articles were selected and assessed again in the second phase. In this step, a full-text review was done to determine the eligible records according to the above criteria. Discrepancies in the final decision were resolved by consensus. The reasons for excluding studies were recorded. The primary outcome was to identify the differences in metabolic patterns between patients with SSc and HC. Secondary outcomes included comparing the differences in metabolic patterns across dcSSc and lcSSc subtypes and cardiopulmonary complications, as well as identifying potential metabolite biomarkers for SSc diagnosis and classification.

### 2.4 Data extraction and result synthesis

Data from each study were manually extracted and transferred into a Microsoft Excel form to include the following variables: (Denton and Khanna, 2017): publication information including first author, year of publication, and study geographic location; (Tsou et al., 2021); patients characteristics including age and sex; (Allanore et al., 2015); sample size; (Pattanaik et al., 2015); sample type; (Elhai et al., 2017); methods used for metabolite identification and analysis, and (Bairkdar et al., 2021) differentially distributed metabolites across comparison groups. Key metabolites features were manually extracted based on statistical significance (*p-*values below a threshold of 0.05 or an area under the receiver-operator curve (AUC) greater than 0.70). These metabolites were then categorized according to the body fluid that was studied (plasma, serum, urine, and exhaled breath) and imported into the software MetaboAnalyst 5.0 for the generation of metabolic pathways enrichment analysis, which provides *p* values adjusted for multiple testing and uses the high-quality SMPDB metabolic pathways as the metabolite set library. There were no methods required for data conversion or the processing of missing summary statistics. Three reviewers (VM, JC, DG) independently extracted the information. Consensus was used to settle any inconsistencies or missing information. Tables were used to present the extracted metabolites.

### 2.5 Quality assessment

The quality of the eligible studies was evaluated using the QUADOMICS evaluation tool (Lumbreras et al., 2008). This scale represents an adaptation of the Quality Assessment of Diagnostic Accuracy Assessment (QUADAS) which assesses the quality of studies on omics-based research. This evaluation tool has 16 items, each of which can be answered with “yes,” “no” or “unclear.” The quality of the included articles was evaluated by three researchers independently, and the discrepancies were resolved by consensus after a comprehensive discussion. The PRISMA checklist for systematic reviews is presented in Supplementary Table S2.

## 3 Results

### 3.1 Study selection

**FIGURE 1:**
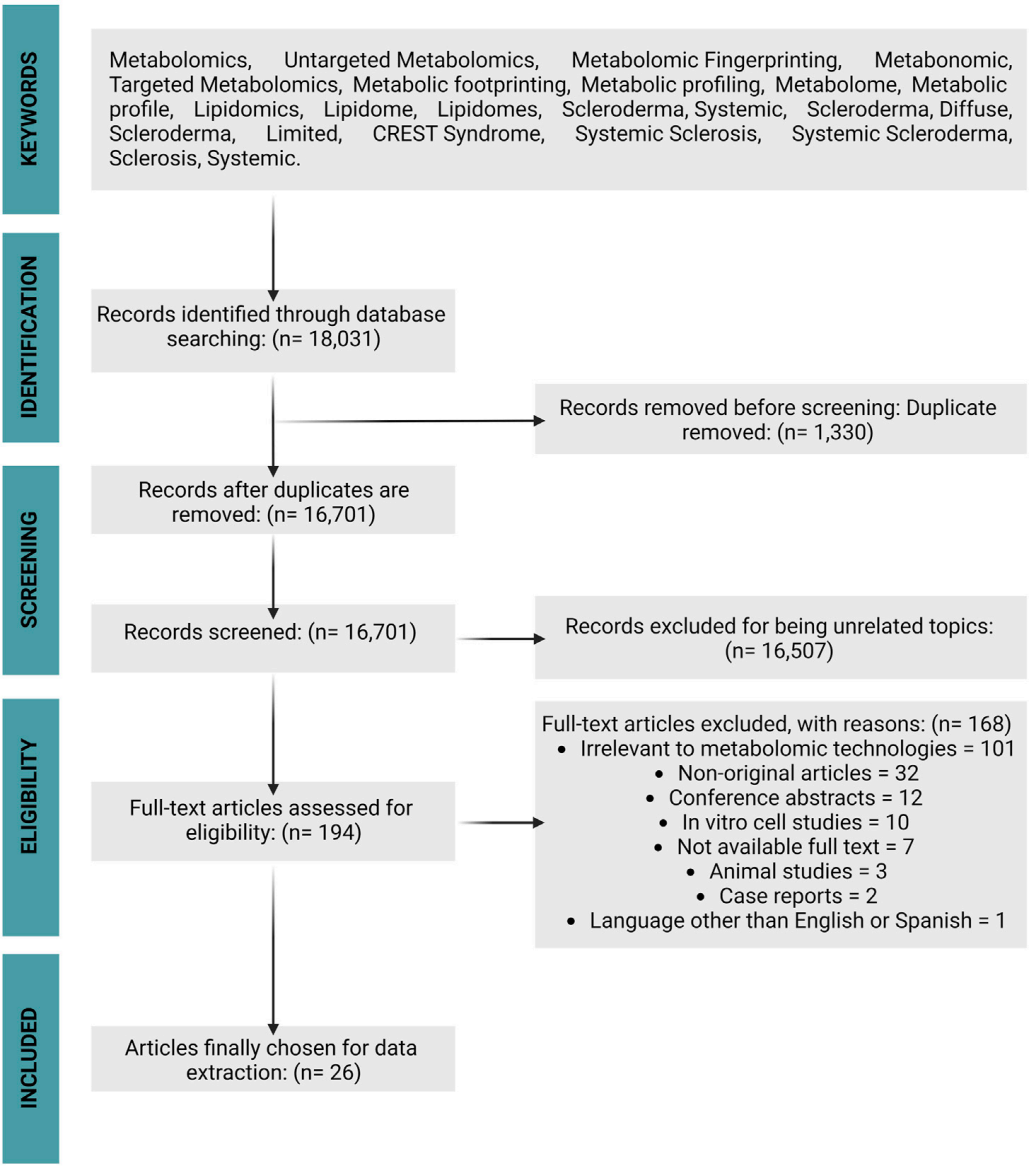
Preferred Reporting Items for Systematic Reviews and Meta-analyses flow chart.

A total of 18,031 records were retrieved from the initial database search, of which 1,330 duplicates were removed by electronic and manual double examination, obtaining a total of 16,701 articles. These were screened by titles and abstracts, excluding 16,507 for being unrelated to the topic of interest. The full text of the remaining 194 articles was fully assessed for eligibility, and finally, 26 articles fulfilled the inclusion criteria. Figure 1 displays the search results and the selection strategy.

### 3.2 Study characteristics

The characteristics of the selected studies are summarized in Table 1. In the selected studies, a total of 2004 individuals were enrolled, including 1,338 patients diagnosed with SSc, most of them within the lcSSc subtype and 666 HC. Most patients diagnosed with SSc included in the studies were women (87.9%). Patients had an average age of 56.3 years. Most of the selected articles were analytical cross-sectional studies (*n* = 23). Twenty-three of the twenty-six studies conducted metabolomics comparisons between SSc cases and HC, two studies compared metabolomic patterns between SSc patients with PAH and SSc patients without PAH (Thakkar et al., 2016; Deidda et al., 2017) and one study compared SSc cases with systemic lupus erythematosus (LES) (Bengtsson et al., 2016). Fourteen studies assessed the metabolome in plasma, eight in serum, two in urine, one in urine and plasma, and one study evaluated in exhaled breath. Most of these studies used high-performance liquid chromatography quadrupole time-of-flight mass spectrometry (HPLC-Q-TOF-MS), high-performance liquid chromatography with fluorescence detection (HPLC-FLD); and to a lesser extent high-performance liquid chromatography quadrupole-linear ion-trap hybrid mass spectrometry (HPLC-QTRAP-MS), high-performance liquid chromatography with on-line UV system. (HPLC-UV) and gas chromatography electron impact mass spectrometry (GC-EI-MS). Proton nuclear magnetic resonance spectroscopy (H NMR), and capillary gas chromatography with flame ionization detection (CGC-FID) were also described.

**TABLE 1:** Characteristics of studies included in the systematic review.

Sample type	Sample size	Gender (M/F)	Age average (years)	Analytical technique	Altered metabolites in SSc vs. HC	Study
Plasma	SSc: 27	4/23	56.9	GC-MS^a^	*Increased:* Nitrate	Neumann Andersen et al. (2000)
	Controls: 27				*Decreased*: NR	
	SSc: 71	10/61	58.5	HPLC-FLD	*Increased:* Homocysteine	Caramaschi et al. (2003)
	Controls: 30				*Decreased*: NR	
	SSc: 15	NR	42.2	GC-MS^a^	*Increased:* Malondialdehyde	Tikly et al. (2006)
	Controls: 13				*Decreased:* NR	
	SSc: 60	4/56	54.6	HPLC-FLD	*Increased:* Homocysteine	Caramaschi et al. (2007)
	Controls: 30				*Decreased:* NR	
	SSc: 40	38/2	58.4	HPLC-MS^a^	*Increased:* NR	Atteritano et al. (2016)
	Controls: 40				*Decreased*: Vitamin D	
	SSc: 59	7/52	56.5	UHPLC-Q-TOF-MS	*Increased:* DL-2-aminooctanoic acid, Diacylglycerol 38:5, 1-(9Z-pentadecenoyl)-glycero-3-phosphate, phosphatidylcholine 36:4, 2,4-dinitrobenzenesulfonic acid, alpha-N-phenylacetyl-l-glutamine	Bellocchi et al. (2018)
	Controls: 28				*Decreased*: NR	
	SSc: 20	3/17	57	UHPLC-Orbitrap-MS	*Increased:* Lauric acid, myristic acid, arachidic acid, carnitine, isovaleryl-carnitine	Ottria et al. (2020)
	Controls: 7				*Decreased*: Octanoyl-carnitine, palmitoyl-carnitine	
	SSc: 42	7/35	59.9	HPLC-TQ-MS	*Increased:* Glutamine, proline, 1-methylhistidine, betaine, methylnicotinamide, asymmetric dimethylarginine	Smolenska et al. (2020)
	Controls: 27				*Decreased:* Tryptophan	
	SSc: 52	8/44	60	HPLC-IM-Q-TOF-MS	*Increased:* Phosphatidylcholine 34:1, 34:2, 34:3; sphingomyelin 33:1, 35:1, 35:2	Geroldinger-Simić et al. (2021)
	Controls: 48				*Decreased:* NR	
	SSc: 52	8/44	60	HPLC-IM-Q-TOF-MS	*Increased:* Kynurenine, dimethylarginine, citrulline, ornithine, phenylacetylglutamine, 1-methylhistidine, 3-methylhistidine	Bögl et al. (2022)
	Controls: 48				*Decreased:* Tryptophan, OH-tryptophan, alanine, lysophosphatidylcholine 22:4a, 22:4b, 20:2; sphingomyelin 34:1, 40:3	
	SSc: 59	7/52	56.5	HPLC-ESI-QTOF-MS	*Increased:* Alpha-N-phenyl acetyl-L-glutamine, butyrylcarnitine, valerylcarnitine, 2-4-dinitrobenzenesulfonic acid, oleic acid, 1-arachidonoylglycerol monoacylglycerol (20:4), monoacylglycerol (20:5)	Fernández-Ochoa ÁQuirantes-Piné et al. (2019)
	Controls: 28				*Decreased:* NR	
Serum	SSc: 10	0/10	47	HPLC-QTRAP-MS	*Increased:* Arachidonoyl-lysophosphatidic acid, sphingosine 1-phosphate	Tokumura et al. (2009)
	Controls: 13				*Decreased*: NR	
	SSc: 68	0/68	67.6	HPLC-TQ-MS	*Increased:* 17β-estradiol, estrone	Aida-Yasuoka et al. (2013)
	Controls: 35				*Decreased*: NR	
	SSc: 19	3/16	55	GC-TOF-MS	*Increased:* Aminomalonic acid, arachidonic acid, arginine, aspartic acid, beta-alanine, cholesterol, inositol-1-phosphate, lauric acid, oleamide, ornithine-1,5-lactam, picolinic Acid, pyroglutamic acid, ribose, succinic Acid, urea, uric acid	Bengtsson et al. (2016)
	Controls: 18				*Decreased*: Alanine, cysteine, lactic acid, malic acid, nonanoic acid, taurine, threonic acid	
	SSc: 37	8/29	58.7	H-NMRS GC-MS^a^	*Increased:* Glutamine, 3-OH-butyrate	Murgia et al. (2018)
	Controls: 20				*Decreased:* Citrate, aspartate, alanine, choline, glutamate, glutarate, glycerate, threonate	
	SSc: 97	16/81	59	HPLC-FLD	*Increased:* Kynurenine	Campochiaro et al. (2019)
	Controls: 10				*Decreased:* Tryptophan	
	SSc: 36	6/30	61.5	UHPLC-Q-TOF-MS	*Increased:* 1-methyladenosine	Meier et al. (2020)
	Controls: 12				*Decreased*: L-tryptophan, L-tyrosine	
	SSc: 30	6/24	46.3	UHPLC-Q-TOF-MS	*Increased:* Vitamin E, alpha-N-phenylacetyl-L-glutamine, L-glutamine, L-isoleucine, phenol, 2-oxoadipic acid, 1-palmitoyl-2-hydroxy-sn, glycero-3-phosphoethanolamine, chenodeoxycholate, indoxyl sulfate, D-quinovose	Sun et al. (2022)
	Controls: 30				*Decreased:* 3b-hydroxy-5-cholenoic acid, 1-stearoyl-glycerol, trans-dehydroandrosterone, 4-nonylphenol, norethindrone acetate, cis-9,10-epoxystearic acid, 16-hydroxypalmitic acid, 2-ethyl-2-hydroxybutyric acid, stearic acid, hexadecanedioic acid, 3 hydroxy caproic acid, androsterone sulfate, benzenebutanoic acid, pregnenolone sulfate, arachidonic acid, dodecanoic acid, palmitic acid, myristic acid, cholesterol 3-sulfate, caprylic acid, Cis-(6,9,12)-linolenic acid, alpha-ketocaproic acid, azelaic acid	
Urine	SSc: 59	7/52	56.5	HPLC-Q-TOF-MS	*Increased:* D-Sorbitol, N-cyclohexylformamide, Ser-Pro-Pro, dihydroxy-1H-indole glucuronide, 2-(2-phenylacetoxy)propinylglycine, alpha-N-phenylacetyl—L glutamine, pyroglutamic acid	Fernández-Ochoa ÁQuirantes-Piné et al. (2019)
	Controls: 28				*Decreased*: N-Methylnicotinamide, proline betaine, creatinine, vinylacetylglycine, N1-methyl-4pyridine-3-carboxamide, N1-methyl-2-pyridine-5-carboxamide, hydroxyprolyl-valine, L-beta-aspartyl-L-Leucine, Hypaphorine, 2-octenoyl-carnitine, decatrienoylcarnitine, 2-nonenoylcarnitine, 2,6-dimethylheptanoyl carnitine, 9-decenoylcarnitine, 9-hydroxydodecenoylcarnitine, undecenoyl carnitine	
	SSc: 11	0/11	51	GC-EI-MS	*Increased:* 15-F-2t-isoprostane	Cracowski et al. (2002)
	Controls: 11				*Decreased*: NR	
	SSc: 43	1/42	54.1	HPLC-UV	*Increased:* 8-isoprostaglandin-F2a	Volpe et al. (2006)
	Controls: 43				*Decreased*: NR	
Exhaled breath	SSc: 46	NR	54	CGC-FID	*Increased:* Ethane	Cope et al. (2006)
	Controls: 21				*Decreased*: Ethanol	

^a^ Detector is not specified in the document.

Increased and decreased metabolites shown in the table correspond to altered metabolites in SSc, patients compared to HC. Abbreviations: SSc, Systemic sclerosis; GC-MS, Gas chromatography-mass spectrometry; HPLC-FLD, High-performance liquid chromatography with fluorescence detection; UHPLC-Q-TOF-MS, Ultra-high-performance liquid chromatography quadrupole time-of-flight mass spectrometry; UHPLC-Orbitrap-MS, Ultra-high-performance liquid chromatography coupled with ion trap mass spectrometry; HPLC-TQ-MS, High-performance liquid chromatography coupled to triple-stage quadrupole mass spectrometer; HPLC-IM-Q-TOF-MS, High-performance liquid chromatography coupled to ion mobility quadrupole time-of-flight mass spectrometry; HPLC-ESI-QTOF-MS, High-performance liquid chromatography coupled to electrospray ionization and quadrupole time-of-flight mass spectrometry; HPLC-QTRAP-MS, High-performance liquid chromatography quadrupole-linear ion trap hybrid mass spectrometry; GC-TOF-MS, Gas chromatography time-of-flight mass spectrometry; H NMRS, proton nuclear magnetic resonance spectrometry; HPLC-QTOF-MS, High-performance liquid chromatography quadrupole time-of-flight mass spectrometry; GC-EI-MS, gas chromatography electron impact mass spectrometry; HPLC-UV, High-performance liquid chromatography with on-line UV, system; CGC-FID, capillary gas chromatography flame ionization detection; NR, Not Reported.

### 3.3 Quality assessment

The results of the methodologic quality assessment by the QUADOMICS tool are summarized in Supplementary Table S3. Because all the studies included in this evaluation were in phase I, the second and 14th QUADOMICS items were not applicable. Overall, the studies meet the majority of the QUADOMICS criterion, indicating that the quality of the included studies is good. All studies described the selection criteria (item 1) and the sample type (item 3), but none met item 12, indicating that the index test findings were interpreted with knowledge of the reference standard. Twenty of the 26 studies found fully comparable data between SSc patients and HC in terms of crucial characteristics including gender and age. Most studies did not avoid overfitting due to the lack of an independent validation set.

### 3.4 Metabolites and metabolic pathways associated with systemic sclerosis

In total, 151 altered metabolites were identified in the selected studies. Since the expression of the metabolites can be influenced by the sample used (Kaluarachchi et al., 2018; Lau et al., 2018) the altered metabolites were detailed according to each fluid.

#### 3.4.1 Plasma

Fourteen studies assessed the metabolome in plasma samples of SSc patients (Caramaschi et al., 2007; Caramaschi et al., 2003; Tikly et al., 2006; Szamosi et al., 2009; McNearney et al., 2010; ichiro et al., 2014; Atteritano et al., 2016; Bellocchi et al., 2018; Ottria et al., 2020; Smolenska et al., 2020; Bögl et al., 2022; Geroldinger-Simić et al., 2021; Fernández-Ochoa ÁQuirantes-Piné et al., 2019; Neumann Andersen et al., 2000). The greatest alterations found are grouped into two chemical classes: amino acids and lipids.

According to amino acids, four studies evaluated plasma Hcy levels in SSc patients vs. HC (Caramaschi et al., 2007; Caramaschi et al., 2003; Szamosi et al., 2009; ichiro et al., 2014). Consistently increased levels of Hcy were found across the studies; however, two of them did not find significant differences in Hcy levels in patients with SSc compared to HC, yet, researchers did find significant differences in the concentrations in patients with vascular and thromboembolic manifestations (Szamosi et al., 2009; ichiro et al., 2014). One study looked at endogenous enkephalin levels in early SSc; nevertheless, they found no significant changes in enkephalin levels between SSc and HC patients, although they did find that low levels were associated with Raynaud syndrome, myositis, and telangiectasias (McNearney et al., 2010). ADMA, Kyn, 1-Methylhistidine, alpha-N-phenylacetyl-L-glutamine, glutamine, proline, citrulline, and ornithine were consistently increased across the studies (Bellocchi et al., 2018; Fernández-Ochoa ÁQuirantes-Piné et al., 2019; Smolenska et al., 2020; Bögl et al., 2022). On the contrary, Trp and alanine were found with downward trends (Smolenska et al., 2020; Bögl et al., 2022).

Lipid content was represented by the classes carnitines, fatty acids (FA), glycerolipids, glycerophospholipids, sphingolipids, and steroids. Short-chain carnitines such as carnitine, butyrylcarnitine, and valerylcarnitine were increased across the studies (Fernández-Ochoa ÁQuirantes-Piné et al., 2019; Ottria et al., 2020). On the contrary, acylcarnitines associated with long-chain fatty acids: octanoyl-carnitine, and palmitoyl-carnitine, were observed with downward trends in SSc patients when compared to HC (Ottria et al., 2020) Regarding FA, high levels of saturated (e.g., lauric acid, myristic acid, and arachidic acid) and unsaturated FA (e.g., oleic acid) were observed (Fernández-Ochoa ÁQuirantes-Piné et al., 2019; Ottria et al., 2020) Similarly, trends in other lipid metabolites were consistently observed. For example, glycerolipids such as DG 38:5, MG 20:4, and MG 20:5 were consistently elevated (Bellocchi et al., 2018; Fernández-Ochoa ÁQuirantes-Piné et al., 2019), yet trends observed in glycerophospholipids, and sphingolipids were not uniform (Bellocchi et al., 2018; Geroldinger-Simić et al., 2021; Bögl et al., 2022). For example, metabolites such as 1-(9Z-pentadecenoyl)-glycero-3-phosphate, phosphatidylcholine 34:1, 34:2, 34:3, 36:4, and sphingomyelin 33:1, 35:1, 35:2 were found to be increased (Bellocchi et al., 2018; Geroldinger-Simić et al., 2021), while lysophosphatidylcholine 22:4, 22:4, 20:2, sphingomyelin 34:1, 40:3 were decreased (Bögl et al., 2022).

Lastly, it was found that vitamin D levels were decreased in patients with SSc, and this deficiency was linked to scleroderma and increased systolic pulmonary artery pressure (Atteritano et al., 2016).

#### 3.4.2 Serum

Seven studies assessed the metabolome in serum SSc patients compared to HC (Tokumura et al., 2009; Aida-Yasuoka et al., 2013; Bengtsson et al., 2016; Murgia et al., 2018; Campochiaro et al., 2019; Meier et al., 2020; Sun et al., 2022). The most significant changes identified in serum are classified into amino acids, lipids, and tricarboxylic acids.

Trends of some altered amino acids in plasma are conserved in serum samples. For example, increased levels of Kyn, alpha-N-phenylacetyl-L-glutamine, glutamine, and ornithine were observed in both fluids (Bengtsson et al., 2016; Murgia et al., 2018; Campochiaro et al., 2019; Sun et al., 2022), meanwhile, Trp, glutamate, and alanine levels remained decreased in serum and plasma (Bengtsson et al., 2016; Murgia et al., 2018; Campochiaro et al., 2019; Meier et al., 2020). Discrepancies were found in levels of aspartic acid. Bengtsson et al. (2016) reported increased levels of aspartic acid in SSc patients when compared to LES patients; however, decreased levels of these metabolites were also observed (Murgia et al., 2018).

The altered lipid content in serum is diverse. In the case of FA and steroids, no clear trends were observed. Some FA, such as lauric acid, 3-OH-butyrate, cholesterol, and chenodeoxycholate, were found to be increased (Bengtsson et al., 2016; Murgia et al., 2018; Sun et al., 2022). On the contrary, FA such as nonanoic acid, azelaic acid, cis-9,10-epoxystearic acid, 16-hydroxypalmitic acid, 2-ethyl-2-hydroxybutyric acid, stearic acid, hexadecanedioic acid, hydroxycaproic acid, dodecanoic acid, palmitic acid, myristic acid and caprylic acid, cis-(6,9,12)-linolenic acid were downregulated (Bengtsson et al., 2016; Sun et al., 2022). Other lipid compounds, such as arachidonoyl-lysophosphatidic acid, were found to be increased in serum from SSc patients (Tokumura et al., 2009). Regarding steroids, 17β-estradiol, estrone, and vitamin E were found to be increased (Aida-Yasuoka et al., 2013; Sun et al., 2022), while androsterone sulfate, pregnenolone sulfate, and cholesterol 3-sulfate were decreased (Sun et al., 2022).

Metabolites indicating altered energy metabolism were identified; however, trends were not consistent. Increased levels of tricarboxylic acid cycle (TCA) metabolites such as succinate were observed (Bengtsson et al., 2016), while citrate and malate levels were found to be decreased (Bengtsson et al., 2016; Murgia et al., 2018). Increased levels of other metabolites such as purines, pyrimidines, and carbohydrates such as uric acid, 1-methyladenosine, picolinic acid, ribose, and D-quinovose were observed (Bengtsson et al., 2016; Meier et al., 2020; Sun et al., 2022) as well as low levels of lactate, choline, taurine, threonate and glycerate in SSc patients (Bengtsson et al., 2016; Murgia et al., 2018).

#### 3.4.3 Urine

Three studies assessed the metabolome in urine (Cracowski et al., 2002; Volpe et al., 2006; Fernández-Ochoa ÁQuirantes-Piné et al., 2019). The most significant changes found in urine samples are chemically categorized into amino acids and carnitines. Urinary levels of alpha-N-phenylacetyl-L-glutamine were found to be increased, as they were in plasma and serum samples (Fernández-Ochoa ÁQuirantes-Piné et al., 2019). Additionally, pyroglutamic acid was upregulated in urine samples in concordance with serum samples (Fernández-Ochoa ÁQuirantes-Piné et al., 2019). Consistent with the results in plasma, urine samples reported decreased levels of acylcarnitines associated with long-chain fatty acids in SSc patients compared to HC (Fernández-Ochoa ÁQuirantes-Piné et al., 2019). Lastly, high levels of 8-isoprostaglandin-F2a were found in urine samples of SSc patients, which were related to more severe lung involvement and active patterns in nailfold video capillaroscopy (Volpe et al., 2006).

#### 3.4.4 Exhaled breath

One study evaluated metabolites in exhaled breath of SSc patients (Cope et al., 2006). They found high breath ethane concentrations which were inversely associated with the diffusing capacity for carbon monoxide, and decreased levels of ethanol concentrations, compared to HC.

#### 3.4.5 Metabolic pathways

**FIGURE 2:**
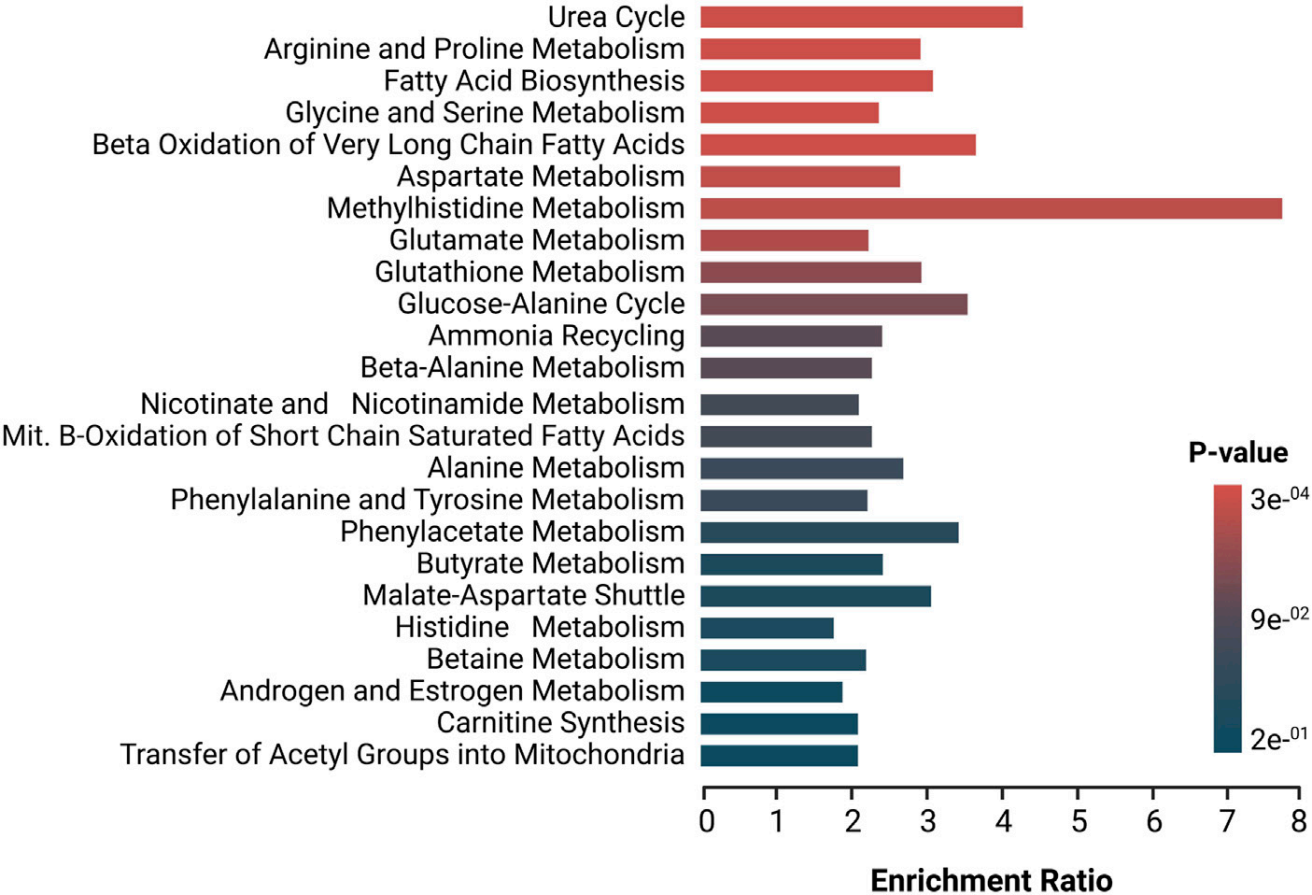
Enrichment analysis of altered pathways in SSc, The significance of pathway alteration is indicated according to the color scale. The bars in red and blue represent the biosynthetic pathways of greater and lesser impact, respectively.

Deregulated metabolites were imported to the MetaboAnalyst platform for the generation of metabolic pathway analyses and SMPDB metabolic pathways were used as a library of metabolite clusters. Figure 2 depicts the altered metabolic pathways in patients with SSc versus HC. Based on the hypergeometric *p*-value test, this software shows whether a metabolic pathway is more strongly represented in the list of compounds. The pathways in red represent the most significant deregulated pathways in SSc patients, while the pathways in blue represent the least significant deregulated pathways in these patients. Several of the amino acids that were found to be deregulated participate in the metabolism of the different pathways that were found to be significantly enriched. As for the urea cycle, several amino acids involved in this cycle, such as glutamic acid, alanine, aspartic acid, ornithine, arginine, urea, glutamine, and citrulline, were found to deregulate, as well as various amino acids involved in the arginine and proline metabolism (e.g., creatinine, glycine, glutamic acid, proline, aspartic acid, ornithine, succinic acid, urea, arginine, and citrulline) several who also participate in the glycine and serine metabolism such as betaine, glycine, alanine, Hcy, arginine, and glyceric acid making them part of the most significant enriched pathways. Similarly, in FA biosynthesis and beta-oxidation of very long-chain FA, several deregulated FA and carnitines were found to be involved in these two pathways (e.g., butyric acid, caprylic acid, myristic acid, dodecanoic acid, caproic acid, and L-carnitine).

### 3.5 Altered metabolism in lcSSc and dcSSc subtypes

Deregulated metabolites in dcSSc versus lcSSc subtypes and HC are described in Table 2. Overall, 7 of the 26 selected studies conducted metabolomics comparisons between dcSSc and lcSSc subtypes in order to discriminate them (Tokumura et al., 2009; Murgia et al., 2018; Fernández-Ochoa ÁQuirantes-Piné et al., 2019; Smolenska et al., 2020; Geroldinger-Simić et al., 2021; Bögl et al., 2022; Sun et al., 2022). Of these, one study evaluated serum samples from SSc subtypes and compared them to HC, finding that levels of sphingosine 1-phosphate were significantly increased in dcSSc patients versus HC (Tokumura et al., 2009). In serum samples from dcSSc patients compared to lcSSc patients, levels of several amino acids, such as valine, glutamate, lysine, and betaine, such as valine, glutamate, lysine, and betaine, carbohydrates, including fructose, glycerol, and glycerate and carboxylic acids, such as acetate and glutarate were found to be significantly increased (Murgia et al., 2018; Sun et al., 2022). On the contrary, levels of glutamine, lactate, and glucose were significantly decreased (Murgia et al., 2018), as well as glycerophosphocholines (Sun et al., 2022). As for plasma samples, dcSSc patients also had higher concentrations of various amino acids and derivatives and phosphatidylcholine species (e.g., Kynurenine, citrulline, ornithine, N(G)-nitro-L-arginine methyl ester (L-NAME), beta-alanine, Phosphatidylcholine 32:0), and lower levels of sphingomyelins and glycerophosphoethanolamines (Smolenska et al., 2020; Geroldinger-Simić et al., 2021; Bögl et al., 2022). On the other hand, urinary metabolites detected in dcSSc patients showed increased levels of amino acids, including L-arogenate and indospicine and N(5-amino-2hydroxybenzoyl)glycine, and decreased levels of carnitines and decreased levels of carnitines in comparison to the lcSSc subtype (Fernández-Ochoa ÁQuirantes-Piné et al., 2019).

**TABLE 2:** Deregulated metabolites in dcSSc vs. HC and lcSSc subtypes.

Sample type	Sample size	Analytical technique	Altered metabolites in dcSSc vs. lcSSc	Study
Serum	dcSSc: 7	HPLC-QTRAP-MS	*Increased:* Sphingosine 1-phosphate	Tokumura et al. (2009)
	lcSSc: 3		*Decreased:* NR	
	Controls: 13			
	dcSSc: 14	H-NMRS GC- MS^a^	*Increased:* Valine, acetate, fructose, glutamate, glycerol, lysine, glycerate, glutarate	Murgia et al. (2018)
	lcSSc: 23		*Decreased:* Sorbitol, glucose, lactate, glutamine	
	dcSSc: 12	UHPLC-Q-TOF-MS	*Increased:* Trans-dehydroandrosterone, betaine, 1-stearoyl-2-oleoyl-sn-glycerol 3-phosphocholine	Sun et al. (2022)
	lcSSc: 18		*Decreased:* 1-palmitoyl-sn-glycero-3-phosphocholine	
Plasma	dcSSc: 21	HPLC-TQ-MS	*Increased:* Sarcosine, beta-alanine, methylnicotinamide, N(G)-nitro-L-arginine methyl ester (L-NAME)	Smolenska et al. (2020)
	lcSSc: 21		*Decreased:* NR	
	dcSSc: 11	HPLC-IM-Q-TOF-MS	*Increased:* Phosphatidylcholine 32:0	Geroldinger-Simić et al. (2021)
	lcSSc: 39		*Decreased:* Phosphatidylethanolamine 38:5, 38:6; sphingomyelin 32:2, 40:4, 30:1	
	dcSSc: 11	HPLC-IM-Q-TOF-MS	*Increased:* Kynurenine, citrulline, ornithine, phenylacetylglutamine	Bögl et al. (2022)
	lcSSc: 39		*Decreased:* Tryptophan, lysophosphatidylcholine 22:4	
Urine	dcSSc: 10	HPLC-ESI-QTOF-MS	*Increased:* L-arogenate, N (5-amino-2hydroxybenzoyl)glycine, indospicine	Fernández-Ochoa ÁQuirantes-Piné et al. (2019)
	lcSSc: 43		*Decreased:* 3-methylglutarylcarnitine, 5-hydroxyindoleacetic acid	

^a^ Detector is not specified in the document.

Increased and decreased metabolites shown in the table correspond to altered metabolites in dcSSc, patients compared lcSSc, or HC. Abbreviations; dcSSc, diffuse cutaneous systemic sclerosis; lcSSc, limited cutaneous systemic sclerosis; HPLC-QTRAP-MS, High-performance liquid chromatography quadrupole-linear ion trap hybrid mass spectrometry; H NMRS, proton nuclear magnetic resonance spectrometry; GC-MS, Gas chromatography-mass spectrometry; UHLPC-Q-TOF-MS, Ultra-high-performance liquid chromatography quadrupole time-of-flight mass spectrometry; HPLC-TQ-MS, High-performance liquid chromatography coupled to triple-stage quadrupole mass spectrometer; HPLC-IM-Q-TOF-MS, High-performance liquid chromatography coupled to ion mobility quadrupole time-of-flight mass spectrometry; HPLC-ESI-QTOF-MS, high-performance liquid chromatography coupled to electrospray ionization and quadrupole time-of-flight mass spectrometry; NR, Not reported.

**FIGURE 3:**
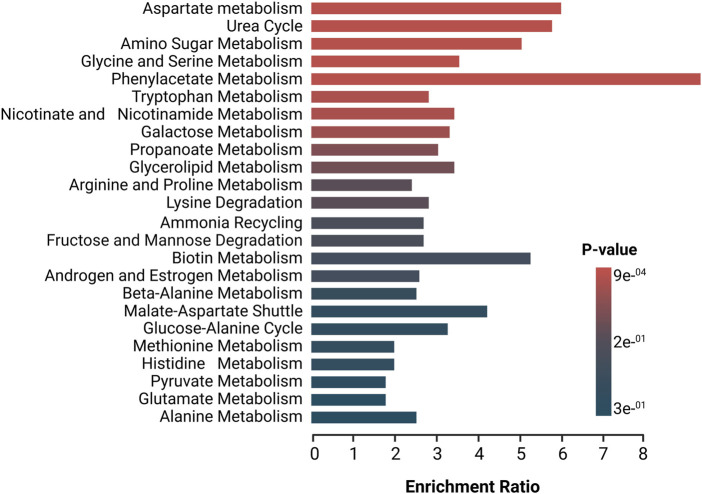
Enrichment analysis of altered pathways in dcSSc. The significance of pathway alteration is indicated according to the color scale. The bars in red and blue represent the biosynthetic pathways of greater and lesser impact, respectively.

In order to further understand these alterations, deregulated metabolites in patients with dcSSc subtype were imported to MetaboAnalyst 5.0 platform for the generation of metabolic pathways analysis, identifying relevant pathways associated with the development of this subtype, using the high-quality SMPDB metabolic pathways as the metabolite set library (Figure 3). Pathways in red represent the most significant deregulated pathways in dcSSc patients. In contrast, the pathways presented in blue represent the least significant deregulated pathways in these patients. The biosynthetic pathways with the greatest impact were the aspartate metabolism, urea cycle, amino sugar metabolism, glycine and serine metabolism, and phenylacetate metabolism. These pathways are associated with alterations in amino acids (e.g., aspartate metabolism, urea cycle, glycine and serine metabolism, and phenylacetate metabolism) with several deregulated amino acids such as beta-alanine, glutamic acid, glutamine, citrulline, ornithine, betaine, and alpha-N-Phenylacetyl-L-glutamine. On the other hand, carbohydrates such as fructose, glyceric acid, and carboxylic acids (e.g., acetic acid) were also involved.

### 3.6 Metabolites associated with pulmonary complications in SSc patients

Of the selected studies, nine evaluated the alterations in the metabolomic profile of pulmonary complications in SSc patients, particularly PAH (Thakkar et al., 2016; Deidda et al., 2017) and ILD (Caramaschi et al., 2003; ichiro et al., 2014; Smolenska et al., 2020; Geroldinger-Simić et al., 2021; Fernández-Ochoa ÁQuirantes-Piné et al., 2019; Sun et al., 2022; Meier et al., 2020). Table 3 describes the identified metabolites associated with pulmonary complications in SSc patients Thakkar et al. (2016) found ADMA levels to be significantly higher and L-arginine levels were significantly lower in SSc-PAH compared with Non-PAH patients. Additionally, studies found that SSc-PAH patients had higher amounts of carboxylic acids (e.g., lactate), and lipoproteins, and lower levels of amino acids, notably L-arginine, in comparison to SSc Non-PAH patients (Deidda et al., 2017). In terms of altered metabolites associated with ILD patients, increased levels of amino acids, such as Hcy, arginine, and valine, and fructosamines derived from branch-chain amino acids were discovered, while lower levels of glycerophosphoethanolamines (e.g., phosphatidylethanolamine 36:3, 38:5, 38:6) and steroids, including androsterone sulfate were found compared to non-ILD (Caramaschi et al., 2003; ichiro et al., 2014; Smolenska et al., 2020; Geroldinger-Simić et al., 2021; Fernández-Ochoa ÁQuirantes-Piné et al., 2019; Sun et al., 2022). One study compared progressive ILD versus stable ILD patients finding increased levels of branched-chain amino acids (BCAAs) and one purine (xanthosine) and decreased levels of adenosine monophosphate in patients with progressive ILD (Meier et al., 2020). Lastly, one study compared urine samples between non-ILD patients and ILD patients, discovering that the latter group also had increased levels of amino acids (e.g., valyl valine, kynurenic acid, L-proline, proline-histidine) (Fernández-Ochoa ÁQuirantes-Piné et al., 2019).

**TABLE 3:** Deregulated metabolites associated with pulmonary complications in SSc patients.

Pulmonary complications	Sample type	Sample size	Analytical technique	Altered metabolites	Study
PAH	Serum	SSc- PAH: 15	HPLC-FLD	*Increased:* Asymmetric dimethylarginine, symmetric dimethylarginine	Thakkar et al. (2016)
		SSc Non-PAH: 30		*Decreased:* L-Arginine	
	Plasma	SSc- PAH: 8	H NMRS	*Increased:* Acetoacetate, Alanine, Lactate, VLDL, LDL	Deidda et al. (2017)
		SSc Non-PAH: 10		*Decreased:* γ-Aminobutyrate, arginine, betaine, choline, creatinine, glucose, glutamate, glycine, histidine, phenylalanine, tyrosine	
ILD	Plasma	SSc-ILD: 65	HPLC-FLD	*Increased:* Homocysteine	ichiro et al. (2014)
		SSc Non-ILD: 151		*Decreased:* NR	
		SSc-ILD: 62	HPLC-FLD	*Increased:* Homocysteine	Caramaschi et al. (2003)
		SSc Non-ILD: 9		*Decreased:* NR	
		SSc-ILD: 18	HPLC-Q-TOF-MS	*Increased:* N-(1-deoxy-1-fructosyl)-Valine, N-(1-deoxy-1-fructosyl)-leucine, N-(1-deoxy-1-fructosyl)-Isoleucine	Fernández-Ochoa ÁQuirantes-Piné et al. (2019)
		SSc Non-ILD: 41		*Decreased:* NR	
		SSc-ILD: 26	HPLC-TQ-MS	*Increased:* Valine, Arginine	Smolenska et al. (2020)
		SSc Non-ILD: 16		*Decreased:* NR	
		SSc-ILD: 14	HPLC-IM-Q-TOF-MS	*Increased:* NR	Geroldinger-Simić et al. (2021)
		SSc Non-ILD: 38		*Decreased:* Phosphatidylethanolamine 36:3, 38:5, 38:6	
	Serum	Stable SSc-ILD: 12	UHPLC-Q-TOF-MS	*Increased:* L-Leucine, L-Isoleucine, Xanthosine	Meier et al. (2020)
		Progressive SSc-ILD: 12		*Decreased:* Adenosine monophosphate	
		SSc-ILD: 19	UHPLC-Q-TOF-MS	*Increased:* L-Glutamine	Sun et al. (2022)
		SSc Non-ILD: 11		*Decreased:* Ile-Ala, Androsterone sulfate	
	Urine	SSc-ILD: 18	HPLC-Q-TOF-MS	*Increased:* Valyl valine, kynurenic acid, L-proline, proline-histidine, quinolinic acid, β-D-glucopyrapyranosil anthranilate	Fernández-Ochoa ÁQuirantes-Piné et al. (2019)
		SSc Non-ILD: 41		*Decreased:* NR	

Increased and decreased metabolites shown in the table correspond to altered metabolites in PAH, patients compared to non-PAH, patients and in ILD, patients compared to non-ILD, patients. Abbreviations: PAH, pulmonary arterial hypertension; ILD, interstitial lung disease; HPLC-SPE, High-performance liquid chromatography with solid phase extraction; H NMRS, proton nuclear magnetic resonance spectrometry; HPLC-FLD, High-performance liquid chromatography with fluorescence detection; HPLC-Q-TOF-MS, High-performance liquid chromatography quadrupole time-of-flight mass spectrometry; HPLC-MS, High-performance liquid chromatography-mass spectrometry; HPLC-IM-Q-TOF-MS, high-performance liquid chromatography quadrupole time-of-flight mass spectrometry; UHPLC-MS, ultra-high-performance liquid chromatography-mass spectrometry; UHPLC-Q-TOF-MS, ultra-high-performance liquid chromatography quadrupole time-of-flight mass spectrometry; NR, not reported.

### 3.7 Metabolites as potential biomarkers

Potential biomarkers identified in the review are described in Table 4. Of all selected articles, five studies assessed the diagnostic capability of biomarkers using AUC, reporting at least one biomarker with an AUC > 0.7 (Thakkar et al., 2016; Murgia et al., 2018; Fernández-Ochoa ÁQuirantes-Piné et al., 2019; Meier et al., 2020; Sun et al., 2022). Of these, three studies evaluated the potential of metabolic biomarkers or panels to diagnose SSc (Murgia et al., 2018; Fernández-Ochoa ÁQuirantes-Piné et al., 2019; Meier et al., 2020), and one study examined diagnostic biomarkers for PAH (Thakkar et al., 2016) finding that serum ADMA levels ≥0.7 μM in PAH patients had a sensitivity of 86.7% and a specificity of 90.0%, two studies evaluated biomarkers for the classification of dcSSc subtype (Murgia et al., 2018; Sun et al., 2022), one study evaluated biomarkers for ILD (Sun et al., 2022), and one study evaluated biomarkers to distinguish progressive SSc-ILD from stable SSc-ILD (Meier et al., 2020). The latter validated their results using an enzymatic assay, obtaining similar results with significantly higher values detected in progressive SSc-ILD patients compared to stable SSc-ILD. These results were also found in another cohort of SSc- ILD patients (Meier et al., 2020).

**TABLE 4:** Potential metabolite biomarkers for diagnosis and classification of SSc.

	Sample type	Analytical technique	Metabolites	ROC curve	Validation	Study
SSc	Serum	H-NMRS	Aspartate	AUC: 0.81 (CL 95% 0.7–0.93)	NR	Murgia et al. (2018)
		GC-MS^a^	Alanine Citrate			
		UHPLC-Q-TOF-MS	L-tryptophan	AUC 0.884 (CI 95% 0.788–0.981)	NR	Meier et al. (2020)
			1-methyl-adenosine	AUC 0.822 (CI 95% 0.705–0.939)		
			L-tyrosine	AUC 0.812 (CI 95% 0.667–0.958)		
	Urine	HPLC-Q-TOF -MS	N1-methyl-4-pyridine-3-carboxamide	AUC: 0.818 (CI 95% 0.709–0.903	NR	Fernández-Ochoa ÁQuirantes-Piné et al. (2019)
			N1-methyl-2-pyridine-5-carboxamide	AUC 0.766 (CI 95% 0.674–0.858)		
			D-sorbitol	AUC: 0.802 (CI 95% 0.688–0.881)		
			2,6 Dimethyl-heptonoylcarnitine	AUC: 0.778 (CI 95% 0.67–0.86)		
	Plasma	HPLC-Q-TOF -MS	Alpha- N-phenylacetyl-L-glutamine	AUC. 0.766 (CI 95% 0.656–0.86)	NR	Fernández-Ochoa ÁQuirantes-Piné et al. (2019)
			1-arachidonoylglycerol monoacylglycerol (20:4) Monoacylglycerol (20:5)	AUC: 0.793 (CI95% 0.687–0.875)		
				AUC: 0.748 (CI 95% 0.64–0.858)		
dcSSc	Serum	UHPLC-Q-TOF-MS	1-Palmitoyl-sn-glycero-3-phosphocholine	AUC 0.650^b^	NR	Sun et al. (2022)
			Trans-dehydroandrosterone	AUC 0.720^b^		
			Betaine	AUC 0.771^b^		
			1-stearoyl-2-oleoyl-sn-glycerol 3-phosphocholine	AUC 0.725^b^		
		H-NMRS GC-MS^a^	Acetate	AUC: 0.84 (CI 95% 0.7–0.98)	NR	Murgia et al. (2018)
			Fructose			
			Glutamate			
			Glutamine			
			Glycerol			
			Glutarate			
PAH	Serum	HPLC-FLD	Asymmetric dimethylarginine	AUC 0.86 (CI 95% 0.7–1.0)	NR	Thakkar et al. (2016)
			Symmetric dimethylarginine	AUC 0.88 (CI 95% 0.74–1.0)		
Progressive ILD	Serum	UHPLC-Q-TOF-MS	L-Leucine	AUC 0.847 (CI 95% 0.695–1.00)	External validation Paris Cohort	Meier et al. (2020)
			L-Isoleucine	AUC 0.826 (CI 95% 0.656–0.997)	Progressive SSc-ILD (*n* = 7)	
			Adenosine monophosphate	AUC 0.785 (CI 95% 0.598–0.971)	Controls (*n* = 27)	
			Xanthosine	AUC 0.771 (CI 95% 0.551–0.9)		
ILD	Serum	UHPLC-Q-TOF-MS	Ile-Ala	AUC 0.807^b^	NR	Sun et al. (2022)
			L-Glutamine	AUC 0.756^b^		
			Androsterone sulfate	AUC 0.778^b^		

^a^ Detector is not specified in the document.

^b^ IC, 95% Not reported.

Abbreviations: SSc, Systemic sclerosis; dcSSc, diffuse cutaneous systemic sclerosis; PAH, pulmonary arterial hypertension; ILD, interstitial lung disease; H NMRS, proton nuclear magnetic resonance spectrometry; GC-MS, Gas chromatography-mass spectrometry; UHPLC-Q-TOF-MS, Ultra-high-performance liquid chromatography quadrupole time-of-flight mass spectrometry; HPLC-Q-TOF-MS, high-performance liquid chromatography quadrupole time-of-flight mass spectrometry; HPLC-FLD, High-performance liquid chromatography with fluorescence detection; ROC, receiver operating characteristic curve; AUC, area under the curve; NR, not reported.

### 3.8 Targeted metabolomics analysis in patients with systemic sclerosis

A total of 10 studies utilized targeted techniques to assess metabolite levels, primarily focusing on amino acids, in patients with SSc (Table 5). In plasma, SSc patients exhibited higher concentrations of metabolites such as Hcy, glutamine, proline, 1-methylhistidine, ADMA, betaine, malondialdehyde, and methylnicotinamide when compared to healthy controls (Caramaschi et al., 2003; Tikly et al., 2006; Caramaschi et al., 2007; Smolenska et al., 2020) Conversely, vitamin D and Trp showed lower concentrations in SSc patients, revealing a distinct trend compared to healthy individuals (Atteritano et al., 2016; Smolenska et al., 2020). Additionally, studies conducted on serum found elevated concentrations of arachidonoyl (20:4)-LPA in SSc patients compared to healthy individuals (Tokumura et al., 2009). Furthermore, there were increased levels of ADMA and symmetric dimethylarginine and decreased levels of arginine observed in SSc-PAH patients compared to SSc Non-PAH individuals (Thakkar et al., 2016). Lastly, urine and exhaled breath investigations demonstrated higher concentrations of 15-F-2t-isoprostane, 8-isoprostaglandin-F2a, and ethane, while the ethanol concentration was reduced in SSc patients (Cracowski et al., 2002; Cope et al., 2006; Volpe et al., 2006). However, comparing metabolites across studies can be complex due to variations in cohorts, analytical techniques, and statistical analysis. Higher concentrations of Hy (11.1–11.8 μmol/L) were observed in the plasma of SSc patients compared to healthy individuals (3.5–6.9 μmol/L), with an approximate increase of 45.41% in Hcy found in SSc patients (Caramaschi et al., 2003; Caramaschi et al., 2007).

**TABLE 5:** Relevant metabolites in SSc patients identified through targeted metabolomics studies.

Sample type	Sample size	Analytical technique	Quantified metabolite	Concentration patients vs. controls (mean ± SD)	Study
Plasma	SSc: 60	HPLC-FLD	Homocysteine	11.8 (10.3–14.5) vs. 6.5 (5.4–8.8) µmol/L^b^	Caramaschi et al. (2007)
	Controls: 30				
	SSc: 15	GC-MS^a^	Malondialdehyde	20.3 vs. 2.48 nmol/L	Tikly et al. (2006)
	Controls: 13				
	SSc: 71	HPLC-FLD	Homocysteine	11.1 vs. 6.9 μmol/L	Caramaschi et al. (2003)
	Controls: 30				
	SSc: 40	HPLC-MS^a^	Vitamin D	25.77 (±12.84) vs. 35.08 (±9.07) ng/mL	Atteritano et al. (2016)
	Controls: 40				
	SSc: 42 Controls: 27	HPLC-TQ-MS	Glutamine	689 (±122.3) vs. 618.4 (±165.3) µmol/L	Smolenska et al. (2020)
			Proline	178.8 (±55.2) vs. 152.5 (±47.3) µmol/L	
			1-Methylhistidine	5.7 (±3.9) vs. 4.1 (±1.5) µmol/L	
			Asymmetric dimethylarginine	0.344 (±0.112) vs. 0.289 (±0.10) µmol/L	
			Betaine	64.8 (±20.8) vs. 52.8 (±17.8) µmol/L	
			Tryptophan	32.5 (±9.6) vs. 40.8 (±12.3) µmol/L	
			Methylnicotinamide	0.312 (±0.166) vs. 0.232 (±0.106) µmol/L	
Serum	SSc: 10	HPLC-QTRAP-MS	Arachidonoyl (20:4)-LPA	2.54 (±0.15) vs. 1.15 (±0.37) nmol/mL	Tokumura et al. (2009)
	Controls: 13				
	SSc- PAH: 15	HPLC-FLD	Arginine	97.28 (±27.4) vs. 117.45 (±26.07) µmol^c^	Thakkar et al. (2016)
	SSc Non-PAH: 30		Asymmetric dimethylarginine Symmetric dimethylarginine	0.76 (±0.14) vs. 0.59 (±0.07) µmol^c^ 0.76 (±0.26) vs. 0.46 (±0.07) µmol^c^	
Urine	SSc: 11	GC-EI-MS	15-F-2t-isoprostane	178 (±32) vs. 95 (±1) µmoles/mmole of creatinine	Cracowski et al. (2002)
	Controls: 11				
	SSc: 43	HPLC-UV	8-isoprostaglandin-F2a	341.7 vs. 147.6 pg/mg creatinine	Volpe et al. (2006)
	Controls: 43				
Exhaled breath	SSc: 46	GC-FID	Ethane	5.27 vs. 2.72 µmol ${ml}^{-1}$ ${CO}_{2}$	Cope et al. (2006)
	Controls: 21		Ethanol	32.5 vs. 76.0 µmol ${ml}^{-1}$ ${CO}_{2}$	

^a^ Detector is not specified in the document.

^b^ IQR, interquartile range.

^c^ Comparison between SSc, patients with PAH vs. SSc, patients without PAH.

Abbreviations: SSc, Systemic sclerosis; SD, standard deviation; PAH, pulmonary arterial hypertension; GC-MS, Gas chromatography-mass spectrometry; HPLC-TQ-MS, High-performance liquid chromatography coupled to triple-stage quadrupole mass spectrometer; HPLC-QTRAP-MS, High-performance liquid chromatography quadrupole-linear ion trap hybrid mass spectrometry; HPLC-FLD, High-performance liquid chromatography with fluorescence detection; GC-EI-MS, gas chromatography electron impact mass spectrometry; HPLC-UV, High-performance liquid chromatography with on-line UV, system; CGC-FID, capillary gas chromatography flame ionization detection.

## 4 Discussion

In this study, we systematically reviewed 26 studies on the metabolomic profiling of SSc and summarized key findings on the dysregulation of major metabolic pathways in SSc, primarily amino acid-related pathways, lipid metabolism, and the TCA cycle. To our knowledge, this is the first systematic review of metabolomic analysis in SSc.

Altered amino acid metabolism was a common finding in analyzed samples of SSc patients, possibly associated with protein synthesis and catabolic processes for energy production (Akram et al., 2011). Increased Hcy levels cause vascular injury by supporting oxidative stress through the production of reactive oxygen species, thus inhibiting antioxidant enzymes, and inducing low-density lipoprotein oxidation in arterial muscle cells (Zhang et al., 2018). Moreover, it induces endothelial dysfunction by inactivating anticoagulant substances (Škovierová et al., 2016). It is noteworthy that both Szamosi et al. (2009) and ichiro et al. (2014) found no difference in Hcy levels between SSc patients and HC; however, significantly higher Hcy concentrations were found in patients with vascular or thromboembolic events in comparison to SSc patients without these manifestations (Szamosi et al., 2009). Likewise, elevated Hcy levels were positively correlated with SSc-ILD (ichiro et al., 2014).

The upregulation of amino acid metabolites such as glutamine, ornithine, proline, and citrulline can lead to the augmentation of collagen synthesis with subsequent fibrosis of the skin and internal organs (Ung et al., 2021). Urea cycle intermediates, such as ornithine and citrulline are involved in proline synthesis (Albaugh et al., 2017). Proline a key component in the synthesis of collagen and the ECM (Karna et al., 2020), is increased in transforming growth factor beta (TGFβ) stimulated fibroblasts, increasing collagen formation and accounting for fibrosis (Schwörer et al., 2020). Glutamine promotes the novo synthesis of proline and sustains collagen synthesis in fibroblasts (Kay et al., 2021). Glutaminolysis is required for the formation of a -ketoglutarate, one of the main collagen I precursors, implying that glutamine metabolism is also important in the development of fibrosis (Ge et al., 2018). Hamanaka et al. (2019) found that the conversion of glutamine to glutamate is required for collagen protein production induced by TGFβ stimulated lung fibroblasts. Additionally, Bernard et al. (2018), tested the role of glutaminolysis in TGF-β1-dependent myofibroblast development and found that TGF-1-differentiated myofibroblasts were compared to controls, glutamate concentrations increased, but glutamine levels decreased, indicating accelerated glutaminolysis. This was linked to TGF- β1 induced mRNA and protein production of the glutaminase (GLS) isoform GLS1 that converts glutamine to glutamate. In this case, extracellular glutamine depletion inhibited TGF-β induced myofibroblast differentiation. Also, glutaminolysis is considered one of the main energy sources for effector T cells and facilitates Th17 proinflammatory phenotype (Cruzat et al., 2018). Several studies have found that the level of Th17 cells in SSc patients is increased compared to HC (Maddur et al., 2012). In SSc, Th17 cells release cytokines that can promote the proliferation and migration of dermal vascular smooth muscle cells inducing endothelial inflammation (Xing et al., 2013). Nevertheless, conflicting roles in Th17 function and fibrosis development have been discussed (Wei et al., 2022). Some studies claimed that Th17 cells induced type I collagen synthesis and secretion to promote fibrosis of murine SSc models (Wilson et al., 2010; Lei et al., 2016; Ramani and Biswas, 2019); however, other researchers propose Th17 cells decrease type I collagen production by dermal fibroblasts (Brembilla et al., 2013; Chizzolini et al., 2018). Additionally, it has been revealed that increased expression of endothelial CCR6, a surface marker of Th17 cell subsets, contributes to the development of SSc vasculopathy (Ikawa et al., 2021). These actions are associated with chronic inflammation and fibrosis, bolstering Th17 cell function in SSc patients.

Furthermore, activation of arginine methyltransferases by inflammation and oxidative stress leads to increased levels of ADMA, the principal endogenous inhibitor of nitric oxide synthase (NOS) (Zhang et al., 2015b). It also leads to an impairment of nitric oxide synthesis, contributing to the augmentation of vasoconstrictor episodes and pathological changes in the vascular system, generating endothelial dysfunction and vascular remodeling (Curtiss et al., 2019). The kynurenine pathway (KP) plays an important role in autoimmune disorders (Boros and Vécsei, 2019). In conditions characterized by inflammation, proinflammatory cytokines such as interferon γ, IL-6, and tumor necrosis factor induce Trp conversion to Kyn by the immune regulatory enzyme indoleamine-2,3-dioxygenase (IDO) (Zou, 2015). Upregulation of IDO enzyme can inhibit mTOR, a regulator of T cell differentiation, and therefore can inhibit effector T cells while promoting regulatory T cells (Treg) (Kurniawan et al., 2020; Sharabi and Tsokos, 2020); however, it has also been demonstrated *in vitro* that Kyn can stimulate mTORC1 activity (Qin et al., 2022). On the other hand, Kyn binds to the aryl hydrocarbon receptor in T cells and dendritic cells promoting the conversion of effector T cells into Treg and promoting IDO induction, therefore establishing a loop to maintain immunotolerance (Lionetto et al., 2021). Additionally, Trp deprivation via IDO mediates cell cycle arrest in the mid-G1 resulting in T cell death and suppression of antigen-specific T cell responses (Krupa and Kowalska, 2021). The Kyn/Trp ratio is a useful marker of IDO activation that reflects the state of immune activation in proinflammatory disorders. Therefore, an increased Kyn/Trp ratio in patients with SSc can be an indicator of increased inflammation and immune system activation.

Alterations in the TCA cycle, the central metabolic pathway for aerobic metabolic processes (Cavalcanti et al., 2014; Arnold and Finley, 2023), were also observed in analyzed samples of SSc patients compared to HC. Increased levels of succinate, a TCA cycle intermediate, were also found. In a study by Tannahill et al. (2013), bone-marrow-derived macrophages stimulated with lipopolysaccharides (LPS) showed that succinate is induced by LPS, impairing propyl hydroxylase (PHD) activity that leads to hypoxia-inducible factor 1-α (HIF-1 α) stabilization and activation, enhancing IL-1b production during inflammation. Therefore, LPS-induced succinate can serve as a signal to enhance IL-1b expression via HIF-1 α. Furthermore, the accumulation of succinate in lung tissue and myofibroblast can contribute to metabolic dysregulation in fibroblasts disrupting PHD activity and enhancing HIF-1 α, promoting the development of lung fibrosis (Wang et al., 2021a). Additionally, Henderson et al. (2020) performed *in vitro* studies in dermal fibroblasts derived from SSc patients and stimulated isolated normal healthy dermal fibroblasts (NHDFs) with TGF-β1 to activate fibrotic pathways and measured succinate levels. After stimulation, they found significantly higher levels of this metabolite in NHDFs, as well as elevated levels of succinate receptor GPR91 in SSc dermal fibroblasts suggesting that succinate released from macrophages can activate fibroblast to undergo fibrotic changes leading to enhanced ECM. Succinate activation of GPR91 has been shown to be important for fibroblast activation and ECM formation in murine intestinal fibrosis and non-alcoholic steatohepatitis (NASH)-associated fibrosis, as well as in fibrotic lung tissue from idiopathic pulmonary fibrosis patients and bleomycin-induced mice (Macias-Ceja et al., 2019; Liu et al., 2020).

On the contrary, citrate levels were significantly decreased. As a TCA cycle intermediate, citrate is crucial for energy production (Iacobazzi and Infantino, 2014). The high consumption of citrate to meet energy charges could explain its low levels, reflecting a reduction in energy availability and increased demand under inflammatory conditions. Various studies have reported decreased levels in samples of patients with autoimmune diseases underlining its importance in immune-mediated inflammatory pathologies (Ouyang et al., 2011; Alonso et al., 2016). Moreover, Yang et al. (2015) demonstrated that a decrease in citric acid accompanied by a decrease in glucose means an increase in energy consumption. Increased glycolysis plays a critical role in fibroblast differentiation and the progression of fibrosis (Zhu et al., 2019). It has been shown that TGF-β1 can cause a rewiring of cellular metabolism, including a shift toward glycolysis, uncoupling from mitochondrial oxidative phosphorylation, and increasing glutamine metabolism (Hewitson and Smith, 2021). In experimental models of SSc, the profibrotic M2 macrophages isolated from bleomycin-induced fibrotic mouse lungs showed increased glycolysis, suggesting its importance in assuring energy efficiency (Xie et al., 2017).

In the case of lipid metabolism, SSc patients showed alterations in carnitines, FA, glycerophospholipids, glycerolipids, sphingolipids, and steroids. Acyl-carnitines play an important role in cellular energy metabolism as a transporter of FA chains into the mitochondria, where long-chain FA are further oxidized (Fielding et al., 2018). Therefore, the downregulation of acyl-carnitines leads to perturbations in fatty acids oxidation (FAO), subsequently increasing FA metabolism (Beger et al., 2018). Furthermore, perturbations of FAO can shift T helper cell differentiation towards a proinflammatory Th17 phenotype (Slack et al., 2015). Additionally, *in vitro* studies have demonstrated that FA accumulation in non-adipose tissues, defined as lipotoxicity, due to FAO inhibition, promotes inflammation, oxidative stress, and fibrosis in renal tubular epithelial cells (Kang et al., 2015a). Moreover, in macrophages, increased FA metabolism can induce a switch to a profibrotic M2 phenotype, playing an important role in fibrosis (Nomura et al., 2016; Wang et al., 2017). In tissues, the healing process depends on whether the initial insult persists or not. If the insult persists, chronic activation of M2 can directly regulate the development and progression of fibrotic lung diseases through the production of chemokines, tissue inhibitor of metalloproteinases, and fibronectin, as well as the capability of M2 to differentiate into fibrocyte-like cells that express collagen, opposite to their primary anti-inflammatory activity through the release of TGF-β, IL10, and arginase, controlling wound healing and tissue regeneration (Braga et al., 2015; Kishore and Petrek, 2021). In the kidney, M2 macrophages induce Th2-type immune responses, secrete large amounts of TGF-β and anti-inflammatory cytokines, transform into myofibroblasts in the injured kidney, inhibit immune responses, and promote wound healing and tissue fibrosis (Wang et al., 2021b).

Regarding sphingolipids, increased levels of sphingosine 1-phosphate (S1P) were observed. Deregulation of S1P in the pulmonary endothelium can lead to vasoconstrictive episodes and vascular remodeling increasing pulmonary vascular resistance (Gluschke et al., 2022). Moreover, S1P influences antigen uptake and presentation by dendritic cells (Arlt et al., 2014). Additionally, the S1P receptor, sphingosine-1-phosphate, modulates early fibrogenesis (Schmidt et al., 2017).

DcSSc and lcSSc subtypes are each characterized by different clinical manifestations, and disease progression (Herrick, 2018); however, the dcSSc subtype is associated with more severe and aggressive organ involvement (Smeets et al., 2020). Therefore, early identification of the disease subtype is imperative to achieve the effectiveness of therapeutic interventions. In this review, analyzed samples of dcSSc patients in comparison with lcSSc demonstrated significantly increased levels of amino acid-related pathways, involved in fibrosis, endothelial dysfunction, and gut dysbiosis. L-NAME, a NOS inhibitor was increased in the dcSSc subtype, suggesting a more severe endothelial dysfunction that could lead to vascular complications (Dooley et al., 2006). As for gut dysbiosis, phenylacetylglutamine (PAG), a gut microbiota-derived metabolite (Teufel et al., 2010), is consistently upregulated in SSc patients. A reduced number of commensal bacteria promotes an excess of substrate favoring PAG formation, suggesting that its deregulation could be an indicator of gastrointestinal involvement (Poesen et al., 2016).

In SSc patients, cardiopulmonary complications are the leading cause of death (Bruni et al., 2021). In this context, screening for PAH and ILD in SSc has emerged as an important consideration. Thakkar et al. (2016) displayed decreased levels of L-arginine, a common substrate of NOS (Cziráki et al., 2020), and increased levels of ADMA, suggesting an association with SSc-PAH. Decreased levels of amino acids with protective effects against endothelial dysfunction and anti-inflammatory effects by inhibition of proinflammatory cytokines, and oxidative stress reduction (Zhong et al., 2003; Hasegawa et al., 2012; Zhao et al., 2018), such as glycine, histidine, and betaine were also found in these patients. Furthermore, elevated levels of low-density lipoprotein in the lungs may lead to lipotoxicity, inducing inflammation and oxidative stress, which causes pulmonary vascular remodeling (Calvier et al., 2022). In SSc-ILD patients, metabolites such as Hcy, proline, glutamine, and BCAAs were elevated, which are involved in the amino acid pathways associated with fibrosis and inflammation. The upregulation of BCAAs can enhance proinflammatory phenotype by activating nuclear factor kappa B in immune cells and over-expression of IL-6 and tumor necrosis factor (Zhenyukh et al., 2017). On the other hand, phosphatidylethanolamine (PE) levels were decreased. Vazquez-de-Lara et al. (2018) demonstrated that PE could attenuate bleomycin-induced lung fibroblast, by decreasing the soluble collagen concentration in mice lungs.

**FIGURE 4:**
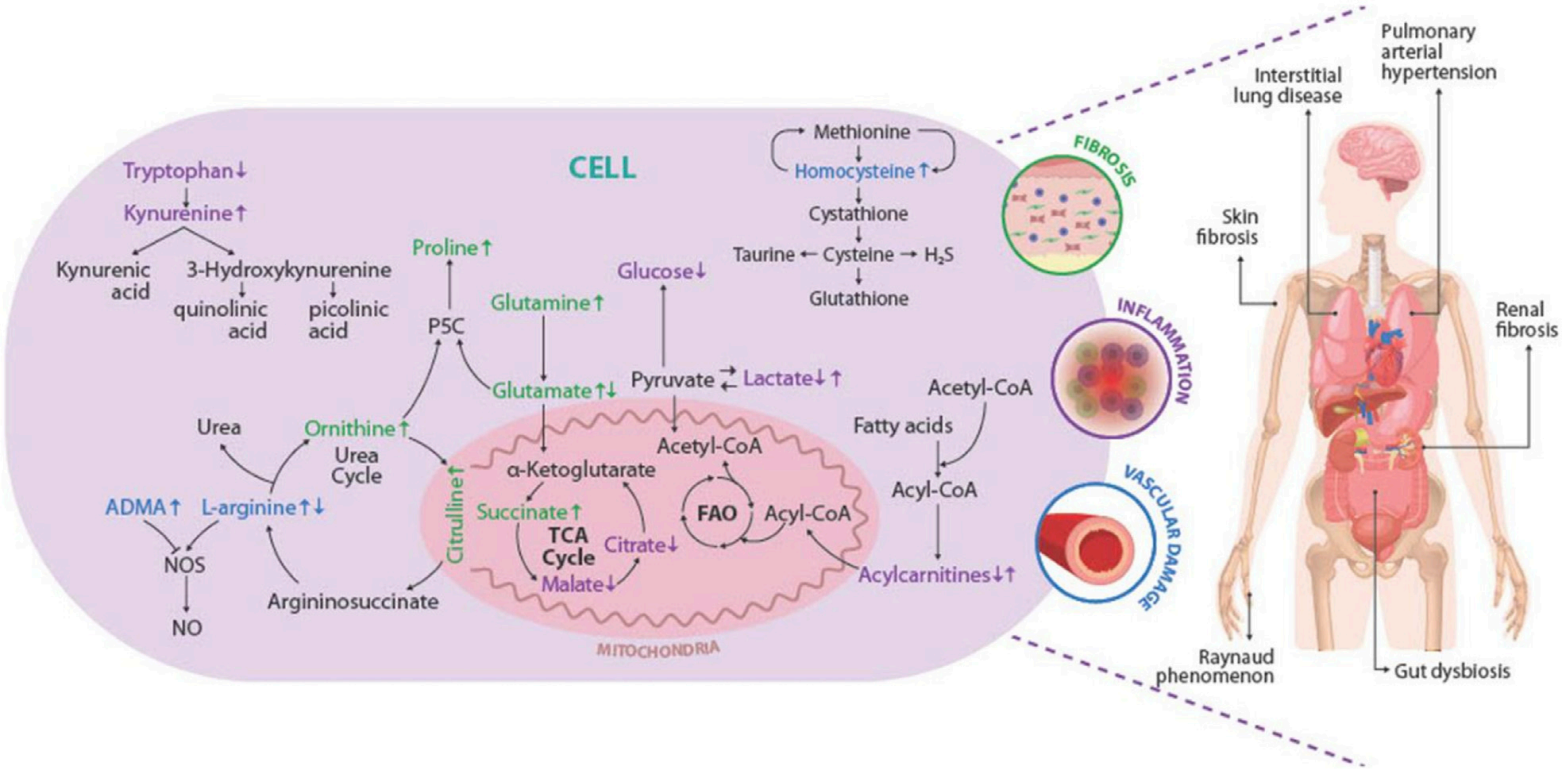
Overview of the deregulations found in metabolites and metabolic pathways and their association with the pathophysiology of the SSc. The main deregulated metabolites and metabolic pathways identified in SSc are represented inside the cell. The metabolites linked with fibrosis are shown in green, those related to the presentation of vascular injury in blue, and those associated with the development of inflammation in purple. Increased metabolite levels are shown by up arrows, whereas decreased metabolite levels are represented by down arrows. Representation of the pathogenic triad of SSc: fibrosis of the skin and internal organs (green), vascular damage (blue), and inflammation (purple). The main clinical manifestations and complications of SSc are represented in the human figure. P5C, sphingosine-1-phosphate receptor; TCA cycle: tricarboxylic acid cycle; FAO, fatty acid beta-oxidation; NO, Nitric oxide; NOS, Nitric oxide synthase; ADMA, Asymmetric dimethylarginine; H_2_S, hydrogen sulfide.

To summarize, Figure 4 depicts a graphic overview of the main deregulated metabolites and metabolic pathways identified in SSc, as well as the role that these could play in the pathophysiology of the disease, leading to the appearance of clinical manifestations and complications associated with the disease.

Metabolomics, as a fast-developing technique in biomedical research, can be used to identify novel biomarkers (Kang et al., 2015b) and as a promising predictive or personalized medicine research technique (Zhou and Zhong, 2022). However, this systematic review has certain limitations, the most significant one is the challenge of comparing the metabolomic acquired across studies due to different limitation factors. Due to the scarcity of available quantitative data, the potential application of meta-analysis is limited, reducing the capacity to make more solid and generalizable conclusions. Variations in sample sources, sample preparation techniques, and metabolite detection methods may be to blame for the heterogeneity of results among studies. Another key constraint is the requirement for further research to validate metabolomics findings in multiple cohorts or independent populations. Validation of results is critical to ensuring the robustness and therapeutic usefulness of the proposed biomarkers. Likewise, most of the studies evaluated were cross-sectional, preventing us from determining a causal association in the metabolic changes associated with SSc. Despite these limitations, metabolomics remains a valuable tool, offering a unique opportunity to understand the metabolic basis of the disease and develop new diagnostic and treatment strategies.

In this review, potential biomarkers were described for the diagnosis of SSc, the identification of the dcSSc subtype, and the identification of primary pulmonary complications such as PAH, and ILD. These potential biomarkers were mainly within amino acids, nucleotides, carboxylic acids, and carbohydrate metabolism. More data are necessary concerning the specificity of biomarkers; as well as external validation studies in other and larger populations; however, we expect that metabolomics will provide more accurate and more validated biomarkers for the detection of SSc.

## 5 Conclusion

The data extracted from the 26 studies showed distinct metabolic profiles between SSc patients and HC and distinct profiles between SSc subtypes, generating new insights for non-invasive prognostic and early diagnostic biomarkers to improve individualized treatment and delay disease progression. Although the metabolic profile can still be affected by a series of other factors, the results obtained suggest the presence of a metabolic fingerprint of the disease. The disrupted metabolite mechanisms identified in this study, mainly, but not exclusively, involving amino acids and lipid metabolism, as well as TCA cycle dysregulation are associated with autoimmune inflammation, vascular damage, fibrosis, and gut dysbiosis, which might be relevant for the development of SSc. Nevertheless, further studies are required to evaluate the role of these alterations in the pathophysiology of the disease, as well as to assess whether these metabolomic networks have potential as treatment targets or as biomarkers not only for diagnosis but also for prognosis and treatment response.
